# Analysis of Heart Transplant Survival Data Using Generalized Additive Models

**DOI:** 10.1155/2013/609857

**Published:** 2013-05-23

**Authors:** Masaaki Tsujitani, Yusuke Tanaka

**Affiliations:** ^1^Department of Engineering Informatics, Osaka Electro-Communication University, Osaka 572-8530, Japan; ^2^Clinical Information Division Data Science Center, EPS Corporation, Osaka 532-0003, Japan

## Abstract

The Stanford Heart Transplant data were collected to model survival in patients using penalized smoothing splines for covariates whose values change over the course of the study. The basic idea of the present study is to use a logistic regression model and a generalized additive model with *B*-splines to estimate the survival function. We model survival time as a function of patient covariates and transplant status and compare the results obtained using smoothing spline, partial logistic, Cox's proportional hazards, and piecewise exponential models.

## 1. Introduction

Cox's proportional hazards model has been proposed based on the relationship between survival and the patient characteristics observed when the patient entered the study [1]. When the values of covariates change over the course of the study, however, a number of theoretical problems with respect to the baseline survival function and the baseline cumulative hazard function need to be solved [2]. Several prognostic models [3–6] have become as widely used as Cox's proportional hazards model for the analysis of survival data having time-dependent covariates. The present study examines the nonlinear effects of the evolution of the covariates over time using penalized smoothing splines.

Cox's proportional hazards model postulates that the hazard at time *t* is the product of two components:

(1) h(t)=h0(t)exp⁡[∑i=1Ibixi],

where **b** = (*b*_1_,…, *b*_*I*_) is a vector of coefficients. The proportional hazards assumption is that the baseline hazard *h*_0_(*t*) is a function of *t* but does not involve the values of covariates. Several prognostic models for heart transplant survival data have been developed using Cox's regression analysis, and the values of all covariates are determined at the time when the patient entered the study [7–9]. However, situations may exist in which the values of covariates change over the course of the study. A time-dependent model uses the follow-up data to estimate the effect of the evolution of the covariates over time. The relative hazard *h*(*t*)/*h*_0_(*t*) then depends on time *t*, and thus the proportional hazards assumption is no longer satisfied [6, 10].

The time-dependent covariates **X**_*l*_^〈*d*〉^ = (*x*_*l*1_^〈*d*〉^,…, *x*_*lI*_^〈*d*〉^) are provided for patient no. *d*, where *x*_*l*1_^〈*d*〉^ is the midpoint of the *l*th time interval. Given the continuous survivor time, piecewise models arise from the partition of the time axis into disjointed intervals. Biganzoli et al. [11, 12] show that by treating the time interval as an input variable in a feed-forward neural network, it is possible to estimate smoothed discrete hazards as conditional probabilities of failure. To apply a generalized additive model (GAM), discretization of one-month or one-week intervals must be applied for the continuous survivor time with time-fixed covariates. However we cannot determine which discretization, one-month or one-week, should be applied; that is, the discretization is not initially unique. In the case of time-dependent covariates **X**_*l*_^〈*d*〉^, *x*_*l*1_^〈*d*〉^ is initially determined as the midpoint of the *l*th time interval for patient no. *d*. It is fairly straightforward to extend the model to survivor data with time-dependent covariates. Furthermore, by regarding a GAM as an extension of a partial logistic model (PLM), the unknown parameters can be estimated by maximizing the partial log-likelihood [13, 14]. 

 We use the Stanford Heart Transplant data, which has been collected to model survival in patients. Although Cox's proportional hazards model is not applicable in the case of time-dependent covariates, the survivor function can be estimated by taking *h*_0_(*t*) in (1) to be the piecewise exponential hazard. Crowley and Hu [7], Aitkin et al. [8], and Lawless [9] used piecewise exponential models and plotted the survival function. Lagakos [15] also examined a graphical technique for assessing covariates in Cox's proportional hazards model based on a permutation of the observed rank statistic. Most previous studies compared the hazard functions to assess the effect of transplantation on survival by fitting pretransplant and posttransplant data separately.

The difficulty is that there is no easily used measure of the difference between the transplanted and nontransplanted groups. Inferences must be based on a comparison of the estimated function. As Aitkin et al. [8] pointed out, there are always dangers in making inferences about the effect of treatment without adequate control groups. We thus provide an analysis that includes pretransplant and posttransplant data simultaneously as time-dependent covariates. It should be emphasized that patients who are not transplanted constitute a control group relative to patients who have undergone heart transplantation by the same covariates.

We use the 1977 version of the data, as given in Crowley and Hu [7], which is for 103 patients. As four of the transplanted patients have incomplete data on the mismatch score, our analysis is based on 99 patients to assess for what values of these covariates, if any, transplantation is likely to prolong survival. More than 30 percent of cases are censored. In these data, survival times are the number of days until death following a heart transplant, as in Lagakos [15]. A distinctive feature of the present problem is that some of the covariates are time-dependent (and possibly random). For example, Table 1 shows the values of covariates for transplant status (i.e., waiting time), age at transplant (in years), mismatch score (as time-dependent covariates), and previous open-heart surgery for patient no. 18. The previous surgery status does not change with time. In order to extend this setting, the covariate for transplant status is taken as an indicator (coded as 0 before the point of transplant and 1 after transplant). All the other time-dependent covariates are treated as being zero before transplant but changing from zero to the actual value of the particular covariate at the time of transplant. Patient no. 18 generated six observations. The proposed methods allow for simultaneous investigation of several covariates and provide estimates of the survival function as well as the significance. 

## 2. Generalized Additive Models

By extending the PLM for the grouped data based on partial likelihood as introduced by Cox [16] and Efron [17], a PLM can be proposed for ungrouped data [13, 14] having time-dependent covariates for the discrete hazard rate *h*_*l*_^〈*d*〉^ of patient no. *d* at the time interval *l*:

(2) PLM: ln⁡(hl〈d〉1−hl〈d〉)=β0+β1xl1〈d〉+β2xl2〈d〉+β3xl3〈d〉+⋯+βIxlI〈d〉.

In recent years, a variety of powerful techniques have been developed for exploring the functional form of effects. Here, GAM with smoothing splines proposed by Hastie et al. [18, 19] will be used by extending the generalized linear model (GLM) in McCullagh and Nelder [20], where the linear predictor in (2) is specified as a sum of smooth functions *s*(*x*) with twice continuously differentiable functions of some or all of the covariates:

(3) GAM: ln⁡(hl〈d〉1−hl〈d〉)=β0+s1(xl1〈d〉)+s2(xl2〈d〉)+s3(xl3〈d〉)+⋯+sI(xlI〈d〉).

The smooth functions in (3) can be represented as

(4) s0(x)=∑j=1q0βjb0j(t),s1(x)=∑j=1q1βq0+jb1j(x),⋮sI(x)=∑j=1qIβqI−1+jbIj(x),

where *q*_1_, *q*_2_,…, *q*_*I*_ are the numbers of knots, and

(5) β=(β0,β1,…,βq0,βq0+1,βq0+2,…,βq0+q1, …,βq0+q1+⋯+qI−1+1,βq0+q1+⋯+qI−1+2, …,βq0+q1+⋯+qI).

For time interval *l* of patient no. *d*, we have the following definitions:

(6) δl〈d〉={1:  patient  no.  d  died  at  time  interval for  the  lth  clinic  visit,0:  otherwise,δl′〈d〉={1:  patient  no.  d  was  censored  at time  interval  l,0:  o.w,vl〈d〉=(δ1〈d〉,δ1′〈d〉,δ2〈d〉,δ2′〈d〉, …,δl−1〈d〉,δl−1′〈d〉)=(0,0,…,0),vl′〈d〉=(vl〈d〉,δl〈d〉)=(0,0,…,0,δl〈d〉),

where **v**_*l*_^〈*d*〉^ is the history of defaults and is censored for the first *l* − 1 time intervals of patient no. *d* and **v**_*l*_^′〈*d*〉^ = (**v**_*l*_^〈*d*〉^, *δ*_*l*_^〈*d*〉^) is the same history extended to include *δ*_*l*_^〈*d*〉^. Using the above model and notation, Tsujitani and Sakon [13] derived the full log-likelihood for all patients

(7) ln⁡L=ln⁡L(β)+∑d=1n ∑l=1ldln⁡p(δl′〈d〉 ∣ vl′〈d〉)

with the partial log-likelihood

(8) ln⁡L(β)=∑d=1n{∑l=1ld−1ln⁡(1−hl〈d〉)+δld〈d〉ln⁡hld〈d〉 +(1−δld〈d〉)ln⁡(1−hld〈d〉)}.

Although ln⁡*L*(**β**) is not a log-likelihood in the usual sense, it possesses the usual asymptotic properties under fairly broad conditions, as proven in Andelsen and Gill [21]. To avoid overfitting, such models are estimated by penalized maximum likelihood

(9) ln⁡L(β)=∑d=1n{∑l=1ld−1ln⁡(1−hl〈d〉)   +δld〈d〉ln⁡hld〈d〉+(1−δld〈d〉)ln⁡(1−hld〈d〉)}+12∑i=1Iλi∫{si′′(t)}2dt,

where *λ*_*i*_ are smoothing parameters that control the trade-off between the fit and the smoothness. The functions *s*_*i*_(*x*) in (9) are represented by the *B*-spline basis functions *b*_*i*_(*x*); see, for details, Tsujitani et al. [14].

Two model-fitting issues remain. The first concerns the selection of smoothing parameter *λ*_*i*_ in (9). The optimum smoothing parameter choice is outweighed by the easy identification of a covariate's functional form as well as the applicability of established inferential methods to short-term survival prediction. In order to select the smoothing parameters, the algorithm developed by Wood [22–24] can be applied by minimizing generalized cross validation (GCV) as an approximation to leave-one-out CV [23]. It should be noted that the leaving-one-out CV is to allow the deletion of only one observation. On the other hand, the ordinal *v*-fold CV divides the data randomly into *v* groups so that their sizes are as nearly equal as possible. This partition should be made to avoid possible biases, as described in Zhang [25]. In many problems, the ordinal *v*-fold CV is, thus, unsatisfactory in several respects for time-dependent covariates. Applying this kind of data structure to the CV algorithm, we obtain insights into how the partition of data should be carried out. A natural extension of the *v*-fold CV algorithm by setting *v* = *n* is to allow the deletion of the patient with several observations; see, for details, Tsujitani et al. [14].

A second issue is the goodness-of-fit test of the model. After choosing the optimum smoothing parameters via the variant *v*-fold CV algorithm, the deviance allows us to test the goodness-of-fit:

(10) Dev=2(ln⁡Lmax⁡−ln⁡Lc),

where ln⁡*L*_*c*_ denotes the maximized partial log-likelihood under some current GAM and the log-likelihood for the maximum (full) model ln⁡*L*_max⁡_ is zero. The deviance (10) is, however, not even approximately an *χ* ^2^ distribution for the case in which ungrouped binary responses are available; see, for example, Collett [26], Landwehr et al. [27], and Tsujitani and Sakon [13]. The number of degrees of freedom required for the test for significance using the assumed *χ* ^2^ distribution for the deviance is a contentious issue. No adequate distribution theory yet exists for the deviance. The reason for this is somewhat technical; for details, see Section 3.8 in Collett [2]. Consequently, the deviance on fitting a model to binary response data cannot be used as a summary measure of the goodness-of-fit of the model. Thus, bootstrapping is applied to the deviance (10) in order to obtain the goodness-of-fit; see, for details, Efron and Tibshirani [28] and Tsujitani et al. [14]. 

## 3. Example 

As an initial model for the Stanford Heart Transplant data, we employ

(11) Model I:  s(x1)+x2+s(x3)+s(x4)+x5.

GCV is only an approximation of leaving-one-out CV. Alternatively the variant *v*-fold CV is leaving-one-out CV based on each of *n* = 99 patients to allow the deletion of the patient with several observations. By using variant *v*-fold CV and GCV for the initial model, the optimum smoothing parameters for GAM are determined as shown in Table 2. By using a backward elimination procedure, we obtain

(12) Model  II:  s(x1)+x2+s(x3)+x5.

The likelihood ratio (LR) statistic based on deviance can be computed to test the significance of spline effects (i.e., nonlinearity). For example, the spline effect of “Midpoint(*x*_1_)” can also be tested by using

(13) Model III:  x1+x2+s(x3)+x5.

By comparing Model  II with III, the reduction in the value of deviance is Δ = 4.59 with 1.85*d*.*f*. This is significant at the 10% level. The spline effect for “Age(*x*_3_)” is not significant. We thus obtain the final optimum GAM

(14) Model IV:  s(x1)+x2+x3+x5

with a variant *v*-fold score of 654.754.

**Figure 1:**
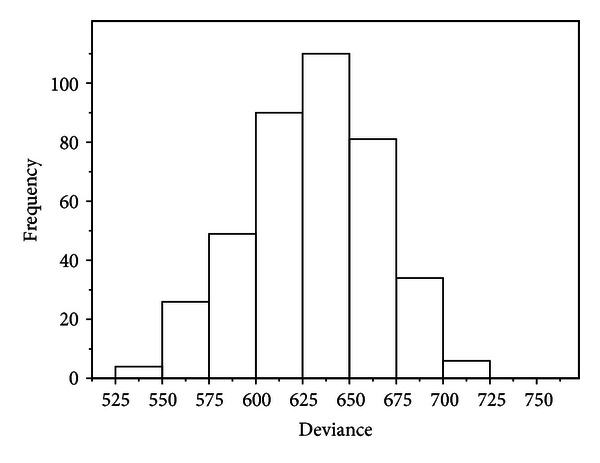
Histogram of the bootstrapped Dev(*b*) for *B* = 400.

Figure 1 shows a histogram of the bootstrapped Dev(*b*) for the optimum model. The bootstrap estimate of the 95th percentile Dev* is Dev* = 685.65. The comparison to Dev = 639.77 of (10) suggests that the model fits the data.

**Figure 2:**
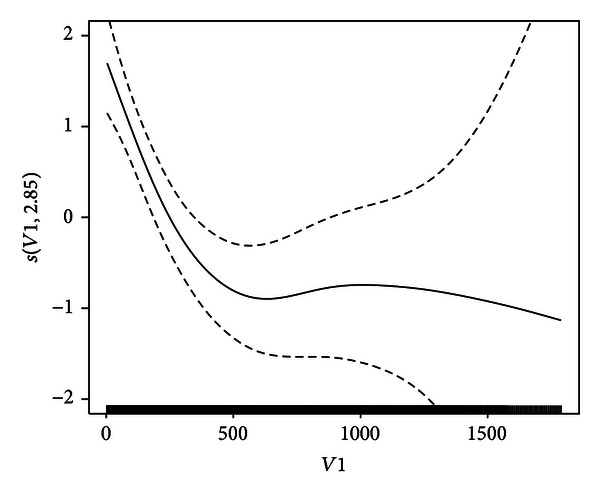
Estimated contribution $\hat{s}(x_{1})$ of *x*_1_ (solid curve) and $\hat{s}(x_{1})\pm 2\text{SD}[\hat{s}(x_{1})]$ (dashed curves).

**Figure 3:**
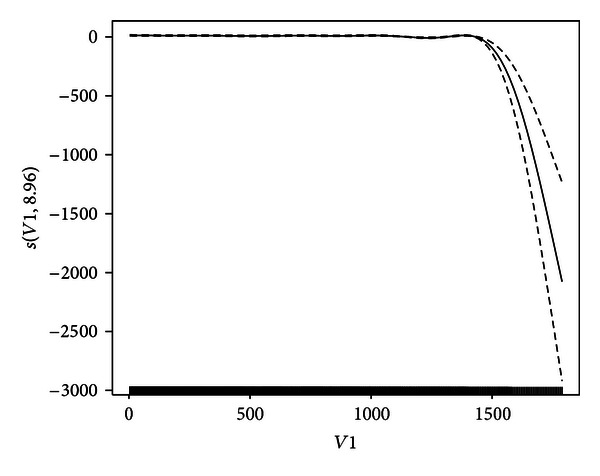
Estimated contribution $\hat{s}(x_{1})$ of *x*_1_ (solid curve) and $\hat{s}(x_{1})\pm 2\text{SD}[\hat{s}(x_{1})]$ (dashed curves) for GCV.

Figure 2 shows the estimated contribution $\hat{s}(x_{1})$ of “Midpoint(*x*_1_)” to $\ln\left\{{\hat{h}}_{l}^{\langle d\rangle }/\left(1-{\hat{h}}_{l}^{\langle d\rangle }\right)\right\}$, together with the ±2 standard deviation (SD) curves for the final optimum Model IV. The spline effects of *x*_1_ are visualized in Figure 2. Figure 2 nicely shows that the spline function *s*(*x*_1_) of dying decreases initially as the midpoint *x*_1_ increases. Subsequently, however, *s*(*x*_1_) is stably maintained after 500 midpoint. For the purpose of comparison, Figure 3 shows the estimated contribution $\hat{s}(x_{1})$ for GCV. From Figure 3, it is clear that the estimated $\hat{s}(x_{1})$ of *x*_1_ is flat until 1500 and then tumbles because of too small smoothing parameter (i.e., overfitting), as shown in Table 2. So variant *v*-fold CV is superior to GCV. The analyses in this example are carried out using library{*mgcv*} in R.

The survival function for our discretized situation is

(15) Pr(l)=∏1≤i≤l(1−hi〈d〉).

The average probability of survival at time interval *l* for patient no. *d* in group *g* can be estimated as

(16) Sg(l)=1nl[g]∑d=1nl[g]Prd[g](l), g=1,2,  l=1,2,…,

where *n*_*l*_^[*g*]^ is the total number of patients at time interval *l* in group *g* and Pr_*d*_^[*g*]^(*l*) is the survival function Pr(*l*) of patient no. *d* at time interval *l* in group *g*; see, for example, Thomsen et al. [29]. 

The data are analyzed to discover which values of the covariates are likely to be of benefit. We compare the results obtained using smoothing spline, partial logistic, Cox's proportional hazards, and piecewise exponential models [7, 8]. The results of fitting the various models are summarized in Table 3. It is clear from Table 3 that

- all covariates for the smoothing spline model are strongly significant (in particular, Crowley and Hu [7] suggested a quadratic effects of age) and
- there is little difference between Cox's proportional hazard model and the piecewise exponential model. It should be noted that binary covariates in the model remain linear.

**Figure 4:**
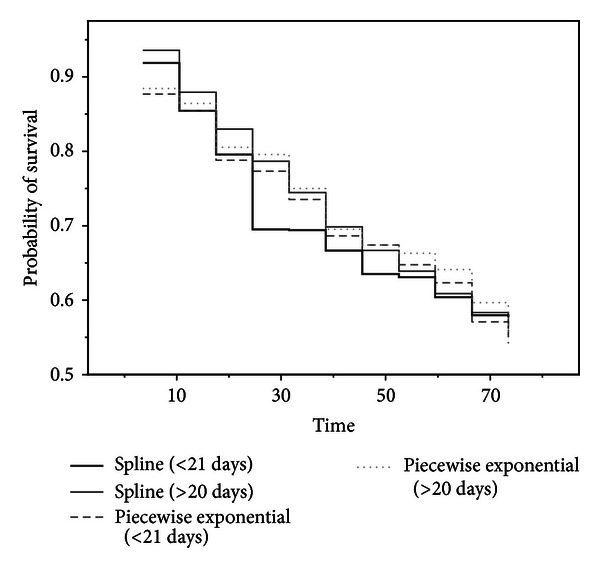
Survival function describing the effect of the waiting time for 41 heart transplanted patients who died.

As shown in Aitkin et al. [8, Figure 2], it is more appropriate to compare survivorship functions if the hazards are not proportional. One point of interest is a comparison of survival experience of transplanted and nontransplanted patients. Our proposal for comparing the survival function is to use the estimated survival function for only 41 heart transplanted patients who died to assess the efficacy of transplantation and the effects of covariates by means of modeling the change in hazard at transplantation by using (15) and (16). Our particular interest is the effect of waiting time on posttransplant survival according to several models. In Figure 4, two time periods are used (group 1: up to 20 days; group 2: longer than 20 days). Figure 4 shows a comparison of the estimated survival function. The estimated survival functions based on the smoothing spline suggest that patients with a short waiting time face a greater early risk than those who had a longer waiting time. However the estimated survival functions based on piecewise exponential models cannot reveal the difference between a short and long waiting times. Our method provides an alternative to Arjas' [10] suggestion of comparing separate estimates of cumulative hazard based on the levels of the waiting time. Although Arjas [10] did not include waiting time as a covariate in Cox's proportional hazard model because of nonproportionality issues, we used transplant status (i.e., waiting time), which is strongly significant for the smoothing spline model according to the results shown in Table 3. 

**Figure 5:**
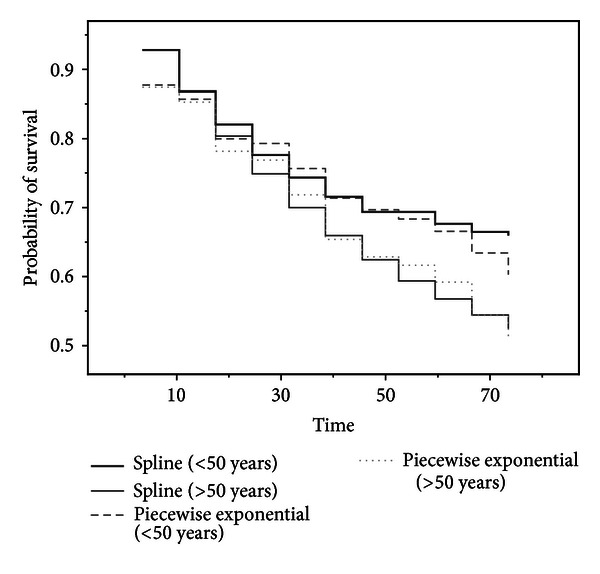
Survival function describing the effect of the age of transplantation for patients with a low mismatch score.

**Figure 6:**
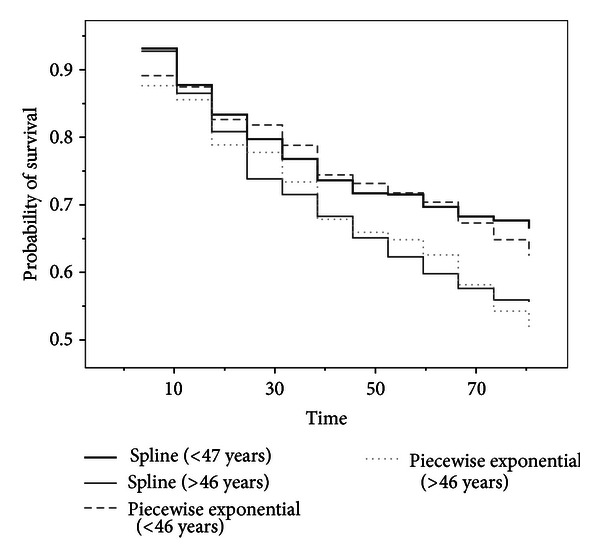
Survival function describing the effect of the age for all 65 transplanted patients.

A fundamentally different type of analysis was suggested by Crowley and Hu [7] to investigate the effect of transplantation with a low mismatch score. They pointed out that transplantation may be beneficial for younger patients only based on regression coefficients for Cox's proportional hazards model, but our conclusion can be derived by graphical analysis as well as significance testing of covariates. Defining a low mismatch score as less than or equal to one for all 29 heart transplanted patients [7], Figure 5 shows a graphical comparison of the estimated survival function for two groups, namely, the younger patients (less than 50 years old at acceptance) and older patients (greater than or equal to 50 at acceptance). From Figure 5, it is clear that older patients face a greater early risk than younger patients; see, for details, Crowley and Hu [7, Chapter 5] with respect to the cutpoints for low mismatch score as less than or equal to one and the younger patients as less than 50 years old. Kalbfleish and Prentice [30, Section  4.6.3] estimated the cutpoint for age, based on all 65 transplanted patients, as 46.2.  Figure 6 shows a graphical comparison of the estimated survival function for two groups, namely, the younger patients (less than or equal 46 years old at acceptance) and older patients (greater than 46 at acceptance). As Kalbfleish and Prentice point out, transplantation is beneficial for younger patients. 

## 4. Conclusion 

We confined our attention to time-dependent covariates. Allowing covariates to vary over the duration of the study not only enabled us to study time-varying risk factors, but also provided a flexible way for modeling censored survival data using penalized smoothing splines. We illustrated the procedures using data of the Stanford Heart Transplant data.

By introducing the maximum likelihood principle into GAM,

- we could visualize the spline effects of the midpoint of the time interval;
- the smoothing parameters could be selected by using variant *v*-fold CV;
- the goodness-of-fit of GAM could be tested based on bootstrapping;
- the estimated average probabilities of survival enabled us to investigate the effect of transplantation with a low mismatch score for two groups, namely, the younger and older patients.

## Figures and Tables

**Table 1:** Covariate values for patient no. 18.

Time interval *l*	Midpoint *x*_1_ (days)	Transplant status *x*_2_	Age at transplant *x*_3_ (years)	Mismatch score *x*_4_	Previous surgery *x*_5_
1	3.5	0	0	0	0
2	10.5	0	0	0	0
3	17.5	1	56	2.05	0
4	24.5	1	56	2.05	0
5	31.5	1	56	2.05	0
6	38.5	1	56	2.05	0

**Table 2:** Optimum smoothing parameters.

Covariates	Variant *v*-fold CV	GCV
Midpoint (*x*_1_)	0.0001	2.75 × 10^−12^
Age (*x*_3_)	0.01	1.30 × 10^−6^
Mismatch score (*x*_4_)	0.01	2.53 × 10^−6^

**Table 3:** *P* values for the significance test of covariates.

Covariates	GAM	Partial logistic	Proportional hazard	Piecewise exponential
Transplant status (*x*_2_)	0.0107	<0.0001	0.0076	0.0081
Age (*x*_3_)	0.011	0.0135	0.0190	0.0199
Previous surgery (*x*_5_)	0.0575	0.0672	0.0830	0.0867
